# Commercial Video Games in School Teaching: Two Mixed Methods Case Studies on Students’ Reflection Processes

**DOI:** 10.3389/fpsyg.2020.594013

**Published:** 2021-01-26

**Authors:** Marco Rüth, Kai Kaspar

**Affiliations:** Department of Psychology, University of Cologne, Cologne, Germany

**Keywords:** game-based learning, reflection, motivation, video game acceptance, commercial video games, guided discovery learning, formal education

## Abstract

Commercial video games are popular entertainment media and part of students’ media reality. While commercial video games’ main purpose is not learning, they nonetheless could and should serve as objects of reflection in formal educational settings. Teachers could guide student learning and reflection as well as motivate students with commercial video games, but more evidence from formal educational settings is required. We conducted two mixed methods case studies to investigate students’ reflection processes using commercial video games in regular formal high school teaching. In a double lesson, 29 students of a 10th-grade biology course (Study 1) and 17 students of a 12th-grade advanced course on history (Study 2) played and discussed a commercial video game related to the current curricular topic. We examined the reflection processes of students in terms of their reactions to the teachers’ game-related statements and questions. Regarding teachers’ statements, students discussed several topics related to game enjoyment and the games’ representation of topic-related content. Regarding teachers’ questions, students discussed multiple goals in each game, how the games represented topic-related content, and how the games could be appropriate for learning. In Study 2, students additionally discussed emotions, stereotypes, violence, and the narrative related to the digital history game. We found that the discussions provided students opportunities to reflect on their game experiences and the current curricular topic as well as to practice media criticism. We further provide quantitative results on students’ perceived topic knowledge, on several facets of their learning motivation, and on their acceptance of video games. Overall, our findings illustrate the educational value of using commercial video games as objects of reflection.

## Introduction

More than 80% of school-aged children are video gamers in the United States (13–17 years; Pew Research Center, 2018), and 15- to 19-year-olds play video games more than an hour each day on average (Bureau of Labor Statistics, 2019). Likewise, about 87% of 12- to 19-year-olds in Germany are video gamers and play games more than an hour per day on average (Pedagogical Media Research Center Southwest, 2019). Thus, for most secondary school students, gaming is a typical leisure activity and part of their everyday life. Beyond their popularity, video games are a cultural good, and the high potential value of using commercial video games in formal education has been articulated for years (e.g., Sandford et al., 2006; van Eck, 2009; Becker, 2017a,b,c; Caldwell et al., 2017; Arnseth et al., 2018). Using commercial video games in school teaching allows teachers to develop students’ media literacy, a key topic of formal education (Squire, 2008) and a cross-sectional and cross-curricular goal relevant to all teachers. In this regard, “the meanings and functions of games cut across formal and informal contexts, but […] this can also be a source of discussion and reflection” (Arnseth et al., 2018, p. 124). Video games include serious games, which are primarily designed and effective for learning and retention of knowledge (Wouters et al., 2013), and commercial off-the-shelf games, which are primarily designed for entertainment purposes and the gratification of players’ intrinsic needs (Ryan et al., 2006). Importantly, there might be a discrepancy between teachers’ assumption that serious games engage students and students’ expectations that playing serious games is like playing commercial games they usually play outside school (Arnseth et al., 2018) so that teachers might provide students the so-called “chocolate-covered broccoli”. In contrast, using commercial video games could allow teachers to authentically address students’ media reality (students’ expectations) and allow them to teach subject-specific topics and the cross-sectional topic of media literacy (teachers’ goals). Accordingly, we argue that the integration of commercial video games into formal school teaching could partly resolve the aforementioned discrepancy, while it is still unclear what forms these integrations might take.

Teachers reported several observations about how using video games in school teaching positively influenced students’ engagement, learning motivation, content knowledge, and subject-specific as well as cross-curricular skills (Huizenga et al., 2017). Notably, positive effects of digital game-based learning are not only based on teachers’ individual observations and assumptions but corroborated by several meta-analytic results (Boyle et al., 2012, 2016; Clark et al., 2016; Hainey et al., 2016). Regarding students’ expectations, the results of a survey comprising 858 secondary school students indicate that they might accept video games in education (Bourgonjon et al., 2010). This is important because, when playing video games as leisure activity, players seem to engage only in lower levels of reflection (Mekler et al., 2018). In formal school teaching, however, students could be triggered to engage in deeper reflection processes after playing commercial video games to address their media criticism skills and media literacy in general.

Commercial video games relate to learning theories (van Eck, 2009; Becker, 2017b), and several features can make them suitable for learning (Gee, 2005; Wilson et al., 2009). For example, players can actively explore and manipulate content, solve problems and challenges, and receive scores or other means of feedback. Still, teachers who decide to use commercial video games should be aware that students could be confronted with inaccurate or wrong information, for example, regarding scientific principles in biology (Schrader et al., 2016) or regarding stories with narrative biases in history (McCall, 2016). Accordingly, commercial video games should not be viewed as mere content providers for formal education. Instead, using commercial video games can trigger student reflection, for instance, via conflicts or competitions in games (Kiili et al., 2011).

While integrating commercial video games in education seems promising, a common key issue is *how* to best align gaming with existing formal educational settings (Mayer, 2019). In line with previous works that highlighted benefits of using commercial video games in education (van Eck, 2009; Becker, 2017c; Caldwell et al., 2017), effective integrations into formal educational settings require appropriate instructional approaches. Arnseth et al. (2018) explicated that teachers need to enable students to reflect on what they do and experience in video games. Similarly, Huizenga et al. (2017) argued that teachers should allow students to interact in the classroom by means of questioning and discussing their game experiences. Indeed, teacher-provided forms of guidance had the largest effect on students’ learning outcomes in digital game-based contexts (Clark et al., 2016). Moreover, meta-analytic findings outline that guided discovery approaches can promote learning and direct reflection processes more than unassisted discovery (Alfieri et al., 2011).

Reflection processes have strong relations to learning and include goal setting, action, and feedback (Seale and Cann, 2000). Reflection processes are also related to playing video games, while playing can be understood as an ongoing cyclic process of acting within the game, getting a reaction, evaluating the reaction, and reflecting on it (Stephenson-Mittlböck, 2012). Reflection processes can take place during gaming (reflection-in-action) and after gaming (reflection-on-action) (Schön, 1987; Brockbank and McGill, 2007b). After gaming, players can reflect on their game experiences by bringing them to mind, thinking about them, and evaluating them against their initial playing strategy, knowledge, or hypotheses (Kiili, 2007). Thereby, students can learn via generalization of experiences or gain a new understanding following a paradigm shift (Brockbank and McGill, 2007c). When playing video games as leisure activity, however, players do hardly reach the corresponding level of reflection to gain new insights, yet they do enjoy reflecting on their game experiences (Mekler et al., 2018). Still, laboratory studies indicate that some game elements can trigger reflections on real-life topics such as mortality (Chittaro and Sioni, 2018). Further, scores in a transfer test were higher if student reflection was guided during digital game-based learning in terms of self-explanations (Johnson and Mayer, 2010), and explanatory feedback, moreover, reduced students’ errors and misconceptions (Moreno and Mayer, 2005). The latter studies provide mostly quantitative (Moreno and Mayer, 2005; Johnson and Mayer, 2010) or qualitative results (Chittaro and Sioni, 2018) based on controlled experimental conditions with single play sessions. Taken together, it thus seems necessary to systematically investigate students’ reflection processes following a single play session in the ecologically valid setting of formal school teaching.

**FIGURE 1:**
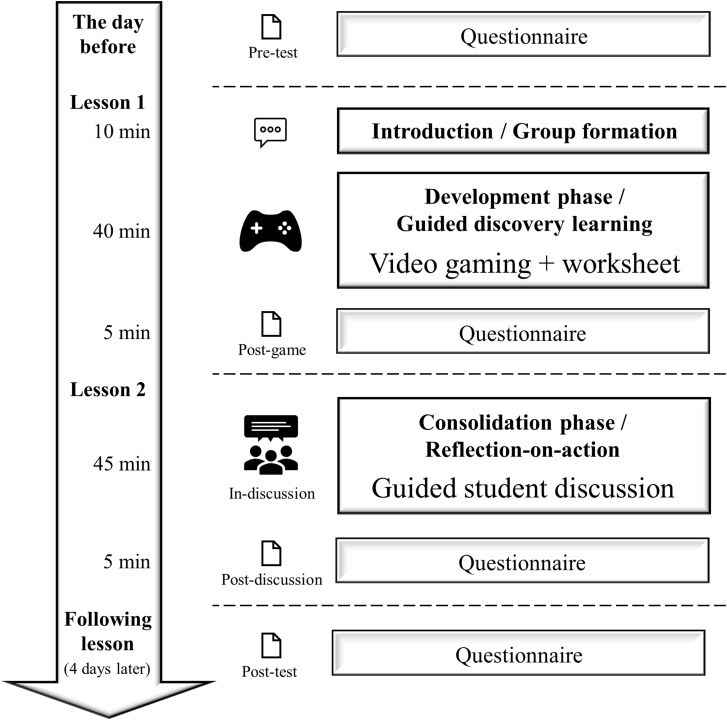
Overview of the study procedure.

In the following, we present two case studies using commercial video games based on a guided discovery approach to teach media criticism and to foster students’ reflection processes. To understand how commercial video games serve as objects of reflection in formal educational settings, we integrated two different commercial video games into two similar formal high school settings. Both settings were regular double lessons, and students had already addressed the topics in previous lessons. In line with curricular guidelines, students’ learning objectives were to analyze topic-related content and to reflect on the topic. To investigate students’ reflection processes, we used measurements the day before the lessons (pre-test), after video gaming (post-game), during a discussion following video gaming (in-discussion), after the discussion (post-discussion), and at the beginning of the following lesson (post-test) (see Figure 1).

In formal educational settings, student discussions allow teachers to debrief students after gaming (reflection-on-action) so that students can share and complement their game experiences and lines of thoughts (Peters and Vissers, 2004). More specifically, teacher-led student discussions can provide students a joint phase of reflection-on-action as well as guidance in terms of reflective support, that is “to assist students in reflecting on the learning process and the knowledge acquired” (De Jong and Lazonder, 2014, p. 375). Worksheets can further structure learning activities and facilitate reflection processes with video games in formal high school teaching (Panoutsopoulos and Sampson, 2012). After gaming, teachers can guide reflection processes by making statements and asking questions related to students’ game experiences, while the overall question is what students discuss:

RQ1: What will students discuss regarding their game experiences in reaction to teachers’ game-related statements and questions?

While worksheets *can* guide student learning, students *have to* pursue games’ goals to make progress in games. Since goals also play a role in the process of reflection and gaming in terms of goal setting and playing strategy (Seale and Cann, 2000; Kiili, 2007), we question which goals students pursue (RQ1a). Moreover, the content in commercial video games is not designed as learning content *per se* (van Eck, 2009; Schrader et al., 2016) and games can transport biased narratives (McCall, 2016) so that it is important to understand how students reflect on the content of games (RQ1b). How students evaluate games’ appropriateness for school teaching (RQ1c) is also of interest, since students’ perceived usefulness of video games can have a strong positive effect on students’ preference for video games (Bourgonjon et al., 2010). Taken together, we address the following research questions:

RQ1a: Which goals of the game will students discuss?
RQ1b: Which content of the game will students discuss?
RQ1c: Which aspects of games’ appropriateness for school teaching will students discuss?

Importantly, effective instruction should include student learning and student motivation (Slavin, 1994). Regarding student learning, both video gaming and student discussions might affect students’ knowledge by means of social interactions between students (Pea, 1993) or by means of students exchanging knowledge and making sense out of individual game experiences together (Palincsar, 1998). We thus question if a gaming phase (RQ2a) and a discussion phase (RQ2b) foster learning via reflection processes (e.g., generalization of experiences or new understandings) in terms of higher students’ perceived topic knowledge. We further differentiate between students’ perceived influence of video gaming and of the discussion (RQ2c):

RQ2a: How will a video gaming phase influence students’ perceived topic knowledge?
RQ2b: How will a guided discussion phase following video gaming influence students’ perceived topic knowledge?
RQ2c: Which influence will students attribute to video gaming, compared to a subsequent guided student discussion, on their perceived topic knowledge?

Regarding student motivation, students were found to be more motivated using video games than in non-game conditions (Clark et al., 2016), which suggests that video games can be promising incentives in formal educational settings. While the main purpose of commercial video games is to fulfill intrinsic personal needs and to elicit enjoyment and motivation (Ryan et al., 2006; Boyle et al., 2012), we consider the issue of providing students “chocolate-covered broccoli” and question if the games still serve their entertainment purpose in formal educational settings. Following Rüth and Kaspar (2020), we therefore address main facets of students’ learning motivation in terms of students’ interest, relevance, competence, satisfaction, and volition. In specific, we here examine students’ learning motivation regarding the lesson’s topic, video gaming, and discussing: first, since video gaming and discussing a commercial video game could trigger learning and reflection processes and provide new insights or perspectives to students, we explore if students’ learning motivation regarding the lesson’s focal topic will change (RQ3a). Second, we assess students’ learning motivation regarding video gaming (RQ3b). Third, we examine students’ learning motivation regarding discussing (RQ3c):

RQ3a: Will students’ learning motivation regarding the lesson’s focal topic (in terms of topic interest, topic commitment, personal relevance of the topic, social relevance of the topic, and volition to learn about the topic) change from pre- to post-test?
RQ3b: Which level of learning motivation regarding video gaming (in terms of game enjoyment, game interest, perceived competence, and satisfaction) will students report at post-game?
RQ3c: Which level of learning motivation regarding discussing (in terms of personal interest, personal relevance, and motivation to participate) will students report at post-discussion?

Finally, decisions on integrating commercial video games into formal educational settings should consider whether students accept video games in such educational settings. Secondary school students’ (age 12–20) preference for using video games in school teaching partly relies on the usefulness and learning opportunities they see in using video games and if they know how to use video games (Bourgonjon et al., 2010). Yet, the survey study of Bourgonjon et al. (2010) suggests to also consider the actual use of video games in formal educational settings, which seems to be neglected in several studies on video game acceptance (Wang and Goh, 2017). Therefore, we here investigate students’ acceptance of video games when these are used in formal school teaching:

RQ4a: Will students’ acceptance of video games in school teaching change from pre- to post-test?
RQ4b: Which general level of acceptance of video games will students report at pre- and post-test?

## Study 1

It has been argued that particularly evolution is a topic that provides many possibilities for scientific misconceptions and misinterpretations (Herrero et al., 2014) so that it is important to foster student reflection on commercial video games addressing evolution. In Study 1, we integrated the commercial video game *Spore* (Maxis, 2008) into a double lesson of biology teaching on evolution. We decided to integrate this video game into formal school teaching based on teacher’s expertise, game reviews, a pedagogical review, and previous game-related conceptual and empirical research: Critics were mostly positive, while gamers had many user discussions, specifically in the year of release (second most discussed PC Game of 2008, Metacritic, 2020a). A pedagogical review outlined Spore as a casual game that allows creating one’s own world, with hardly any consequences of one’s mistakes (Spielbar, 2020a). Previous research criticized Spore conceptually for how it presents evolution and that playing it could result in misconceptions about evolution (Bohannon, 2008; Bean et al., 2010; Ching, 2012; Schrader and McCreery, 2012; Schrader et al., 2016). Schrader and McCreery (2012) concluded that “for many educators, SPORE represents a failure to build serious games”, but it “provides an interesting case to examine learning with a game” (pp. 18–19). In other words, that Spore represents the scientific topic of evolution does not make it a serious game, and unlike for serious games, learning the content that Spore conveys is even not recommended. Still, learning with Spore might be promising when teaching material or student discussions complement the game experiences: A case study found a higher increase in students’ examination scores and more engagement with course material when students played Spore along with other teaching materials compared to students who received traditional instruction in a regular upper-level college class (Poli et al., 2012). Herrero et al. (2014) highlighted several main learning principles included in Spore and provided qualitative evidence on student learning in an extracurricular workshop in which students discussed. Overall, integrating Spore into formal school teaching constitutes an interesting case to investigate how students discuss the topic evolution and reflect on their game experiences.

### Materials and Methods

#### Participants

The sample comprised 29 students from a 10th-grade biology course at a German comprehensive school (high school level) (*M*_age_ = 15.52, *SD*_age_ = 0.63; 16 female). In the analyses, differences in sample size are due to some students being absent at some times of measurement (for an overview, see Supplementary Table 1). Based on a 5-point scale including verbal markers (1 = “never”, 5 = “very frequently”) and ratings from 26 students present at pre-test, students reported to play video games occasionally in their leisure time (*M* = 2.65, *SD* = 1.38, 26.93% never) but seldom in school teaching (*M* = 1.69, *SD* = 0.68, 42.31% never), about evolution (*M* = 1.27, *SD* = 0.72, 84.62% never), and the game Spore (*M* = 1.12, *SD* = 0.59, 96% never, 4% frequently).

#### Design and Procedure

We used a one-group pre–post mixed methods design and collected data at five time points: the day before the lesson (pre-test), during the lesson (post-game, in-discussion, post-discussion), and at the beginning of the following lesson that was 4 days later (post-test). We depict the procedure in Figure 1 as well as the complete measurement plan including all dependent variables, time points, and references to individual research questions in Supplementary Table 1. In a semistructured interview before the study, the biology teacher stated personal interest in video games and some experience with using video games in school teaching. Similar to Rüth and Kaspar (2020), the collaboration with the teacher was initiated via a broad announcement on using video games in formal school teaching in a regional network of teachers.

We employed a strong program of triangulation (Flick et al., 2012) by combining quantitative and qualitative methods (methodological triangulation) and data (data triangulation). On the one hand, we audiorecorded and transcribed the discussion phase (RQ1a–c) and digitized students’ responses to open questionnaire items (RQ2a–c). On the other hand, in the questionnaires, we also used symmetric direct self-rating scales with five levels to measure students’ perceived topic knowledge (RQ2a and RQ2b), learning motivation (RQ3a–c), and acceptance of video games (RQ4a and RQ4b). We used direct ratings to reduce acquiescence response bias (Saris et al., 2010) and to provide students intuitive self-ratings. Each scale was accompanied by verbal markers (e.g., “not at all”, “rather not”, “moderately”, “rather”, and “very”). All questionnaires were in paper–pencil format. Similar measures were used to evaluate the integration of another commercial video game into formal school teaching (Rüth and Kaspar, 2020).

The study took place in a regular double school lesson lasting 120 min. In the first lesson, the teacher shortly outlined the lesson plan, assigned students to groups of four, and provided students with spoken and written (worksheet) task instructions. All groups played the video game for about 40 min, while the teacher ensured that each student played for a similar duration (10 min) and completed a worksheet (development phase). In the second lesson (consolidation phase), the teacher introduced the discussion phase by asking students to rate three different statements about the game and then moderated a semistructured discussion (guided student discussion).

During the double lesson, two carefully instructed observers filled out a structured observation protocol for event sampling. Following Rüth and Kaspar (2020), the purpose of this protocol was to document organizational (e.g., time delays or technical issues) or behavioral aspects (e.g., inattentive or demotivated students), which might be of relevance to assess the validity of our data. According to the observation protocols, some students were late or loud at the beginning of the first lesson but appeared very motivated and concentrated during video gaming, with remaining conversations being only about game experiences. Further, when the double lesson was over, students would have liked to continue playing the game.

#### Teaching Materials

Teaching materials consisted of a lesson plan, worksheets, and teaching guidelines for the gaming phase and the discussion phase. The worksheets and the discussion were meant to foster student reflection by asking them to think about the game’s goals and to which degree the game addresses or simplifies topic-related aspects, takes a specific viewpoint on the topic, or presents evolutionary processes wrongly. We developed the teaching materials in close collaboration with the teacher.

We used the commercial video game Spore (Maxis, 2008; age rating of 12) and left its original game narrative unchanged: Students first watched the cinematic intro and played the build-in tutorial as well as the first two out of five game phases. In phase 1 (emergence of living cells), players control a simple creature, experience that the environment of the game can be beneficial or harmful for their creature, and choose one out of three diets (carnivore, herbivore, or omnivore) for their creature. In phase 2 (evolution to terrestrial creatures), players face more complex appearances and behaviors than in phase 1, since creatures gain lower extremities and can join a group of creatures, respectively. In both phases, players collect experience points that allow them to modify their creature. Each group of students played the game using a laptop computer and an external computer mouse. The repetitive background sound in the game was reproduced by a pair of speakers in the front of the classroom.

#### Measures

##### Guided student discussion (RQ1)

At the start of the discussion phase, the teacher triggered students’ reflection-on-action by asking them to react to three statements: “Playing the game Spore was fun”, “The game vividly illustrates evolutionary processes”, and “The game Spore should be used in biology lessons”. Following each statement, students positioned themselves on an imaginary scale in the classroom ranging from 0 to 10 (RQ1). During the subsequent semistructured discussion, the teacher asked one question about the game’s goal (RQ1a), three questions addressing the game’s content (RQ1b), and two questions addressing games’ perceived appropriateness for school teaching (RQ1c). Question wordings are depicted in Table 1.

**TABLE 1:** Content of the semistructured discussion phase in Study 1.

Category and subcategories	*n*	Teacher’s questions and students’ example statements
**Game’s goals**		What is the general goal of the game? What has allowed or hindered you to reach it?
Development of the creature	2	“The goal of the game was, as a carnivore, to eat a lot of meat and to keep growing, to evolve, or, as an herbivore, to eat many plants, again to evolve and to become bigger” (S17)
Becoming the dominant species	1	“Trying to become more and more dominant and to ally with species within the own environment, or to displace them and to try becoming the most dominant species on the planet” (S5)

**Game’s content**		Are the evolutionary factors mutation, recombination, and selection represented in the game? If so, how? If not, why not? At which points in the game were, in favor of the gameplay, evolutionary processes represented differently or in a simplified way? Were there game scenes, in which evolutionary processes were perhaps even represented incorrectly?
Content-based criticism	3	“For example, when one bought these body parts, that was deliberately changed to have fun” (S10) “Mutation did not take place randomly […] That was the factor, which was changed due to game enjoyment and technical reasons” (S5)
Content identification	3	“Selection I would say […] it happened that one of us died twice in a row. The following time, we said ‘okay, that was not that beneficial, we have to do it differently”’ (S5) “At the beginning, without any defense or body parts for defense, one died much faster. Thus, if one had not put those parts on, one would not have survived” (S22) “At the start, mutation took place and the creature evolved and has thereby adapted. And that leads from mutation to recombination” (S8)

**Games’ appropriateness for school teaching**		Which changes would you make to the game if you were an evolutionary biologist? To what extent do you think video games are suitable for teaching biological knowledge?
Constructive critique	8	“I would design it much livelier, much more realistic” (S7) “I would completely remove the creature editor and keep everything random” (S27) “I would make the evolutionary factors more clearly” (S8)
Games’ potentials	2	“I think that learning with video games is very good in general, because it is not that boring as writing in school. I think one can learn much better with games, because one is keener on learning, and games really show that. And if there is only someone in front and explains it, it is probably more difficult for that person than for a game, which can really visualize it” (S10) “I would agree with S10 that it is rather good that one can visualize such things more easily with games. […] I would rate this a little higher with respect to learning, if it is well done, than, for instance, a documentary” (S5)
Games’ limitations	2	“One aspect one should care about during game development is that the content is disseminated correctly and that there are no errors included, because of which content is disseminated incorrectly” (S5) “What would be missing for me are all those technical terms. That is all well and good, but what is the use of it, if I have no idea what it is called?” (S7)

*Categories and example statements are based on responses of 11 students, while in total, 26 students participated in the discussion.*

##### Students’ perceived knowledge about the topic evolution (RQ2)

We assessed how students perceived the influence of gaming (RQ2a) and discussing (RQ2b) on their knowledge about evolution via self-ratings. At pre-test (the day before the lesson), post-game, and post-test (following lesson, four days later), we asked “How highly do you rate your current knowledge of the topic evolution?” (self-rated topic knowledge). In addition, regarding the specific influence of gaming on topic knowledge (RQ2a), we asked “How much did playing the game on the whole support you in increasing your knowledge of the topic evolution?”, and students also provided open responses (“Please describe in your own words how playing the game influenced your knowledge of the topic evolution.”) at post-game and post-test. In order to assess the specific content that students learned by video gaming, they were asked to recall up to three aspects at post-test (“What did you learn by playing the game? Please write down up to three aspects.”). Regarding the perceived influence of the guided discussion on topic knowledge (RQ2b), students provided self-ratings (“How much did you learn about the topic evolution during the discussion?”) and open responses (“Please describe in your own words how the discussion influenced your knowledge of the topic evolution.”) at post-discussion. Finally, we compared the perceived influence of gaming versus discussing on topic knowledge (RQ2c) by means of open responses at post-discussion (“Please describe in your own words how playing the game in comparison with the discussion influenced your knowledge of the topic evolution.”).

##### Students’ learning motivation regarding lesson’s topic, gaming, and discussing (RQ3)

Regarding the lesson’s topic (RQ3a), we assessed students’ learning motivation in terms of interest in the topic, topic commitment, personal relevance of the topic, social relevance of the topic, and volition to learn about the topic. For item wordings, see Table 2. Learning motivation regarding video gaming was operationalized in terms of game enjoyment, game interest, perceived competence, and satisfaction (RQ3b). Item wordings are depicted in Table 3. Learning motivation regarding discussing was operationalized in terms of personal interest, personal relevance, and motivation to participate in the discussion (RQ3c). Items are depicted in Table 4.

**TABLE 2:** Influence of lessons on students’ learning motivation regarding lessons’ topic in both studies.

	Study 1					Study 2				
Measure	Pre-test *M* (*SD*)	Post-test *M* (*SD*)	*t*	*p*	*d*	Pre-test *M* (*SD*)	Post-test *M* (*SD*)	*t*	*p*	*d*
Topic interest: “How interested are you in [topic]?”	3.67 (1.02)	3.33 (0.80)	−1.67	0.110	0.37	4.25 (0.86)	4.38 (0.72)	0.81	0.432	0.20
Topic commitment: “How much are you concerned with [topic] in your leisure time?”	1.57 (0.81)	1.95 (0.86)	1.79	0.088	0.39	2.50 (0.73)	2.75 (0.86)	1.46	0.164	0.37
Personal relevance of the topic: “How important is [topic] for you?”	2.95 (0.86)	3.29 (0.72)	1.92	0.069	0.42	3.75 (0.77)	4.00 (0.73)	1.46	0.164	0.37
Social relevance of the topic: “How important do you think [topic] is for other people?”	3.52 (0.68)	3.24 (0.62)	−1.37	0.186	0.30	3.31 (1.01)	3.31 (0.95)	0.00	>0.999	0.00
Volition to learn about the topic (three items; Study 1: α = 0.78, Study 2: α = 0.66): e.g. “How important to you is learning about [topic]?”	3.37 (0.77)	3.33 (0.57)	−0.18	0.863	0.04	4.13 (0.53)	4.02 (0.65)	−0.68	0.510	0.17

*Values depict mean (standard deviation) of 21 biology students (Study 1) and 16 history students (Study 2). [topic] is a placeholder for “evolution” (Study 1) and “First World War” (Study 2). Statistical results refer to dependent t-tests comparing the means at pre- and post-test.*

**TABLE 3:** Students’ learning motivation regarding video gaming in both studies.

	Study 1	Study 2
Measure	Post-game *M* (*SD*)	Post-game *M* (*SD*)
Game enjoyment (four items adapted from Reinecke et al., 2012; Study 1: α = 0.91, Study 2: α = 0.94): e.g. “How much did you enjoy playing the game?”	3.65** (0.92)	3.81** (1.06)
Game interest: “How interested are you in the game?”	3.81** (1.33)	4.00** (1.17)
Perceived competence (five items adapted from the Player Experience of Need Satisfaction questionnaire by Ryan et al., 2006; Study 1: α = 0.71, Study 2: α = 0.94): e.g. “How successful did you feel while playing the game?”	3.94*** (0.57)	3.75** (0.99)
Satisfaction: Game preference		
Game recommendation: “How much would you recommend the game to your friends?”	3.04 (1.11)	3.59* (1.06)
Game preference for school teaching: “How much would you like to play the game again in school teaching?”	4.35*** (1.06)	4.12** (1.36)
Game preference in leisure time: “How much would you like to play the game again in your leisure time?”	3.12 (1.37)	3.35 (1.41)
Satisfaction: Game evaluation		
Game graphics: “How much do you like the graphics in the game?”	2.77 (0.95)	3.88** (1.05)
Game music: “How much do you like the music in the game?”	3.00^a^ (0.76)	4.18*** (1.07)
Overall game rating: “How much do you like the game overall?”	3.96*** (0.87)	3.94**^b^ (1.24)
Game’s appropriateness for school teaching: “How much do you think the game is appropriate for use in school teaching?”	3.65** (1.09)	3.71* (1.21)

Values depict mean (standard deviation) of 26 biology students (Study 1) and 17 history students (Study 2). Asterisks indicate the results of one sample *t*-tests comparing the mean with the scale’s midpoint of 3. ^*a*^*n* = 15; ^*b*^*n* = 16. *p < 0.05, **p < 0.01, ***p < 0.001.

**TABLE 4:** Students’ learning motivation regarding discussing in both studies.

	Study 1	Study 2
Measure	Post-discussion *M* (*SD*)	Post-discussion *M* (*SD*)
Personal interest in the discussion: “How interesting was the discussion about [topic] for you?”	3.00 (1.06)	3.88*** (0.78)
Personal relevance of the discussion: “How relevant was the discussion about [topic] for you?”	3.42* (0.90)	3.94*** (0.75)
Motivation to participate in the discussion: “How much were you motivated to participate in the discussion about [topic]?”	2.96^a^ (1.17)	3.41 (1.06)

Values depict mean (standard deviation) of 26 biology students (Study 1) and 17 history students (Study 2). [topic] is a placeholder for “evolution” (Study 1) and “First World War” (Study 2). Asterisks indicate the results of one sample t-tests comparing the mean with the scale’s midpoint of 3. ^*a*^*n* = 25. *p < 0.05, ***p < 0.001.

##### Students’ acceptance of video games (RQ4)

At pre- and post-test, students rated their acceptance of video games in school teaching (RQ4a). We also explored students’ acceptance of video games as a leisure activity and as significant part of life (RQ4b). Items are depicted in Table 5.

**TABLE 5:** Students’ acceptance of video games in both studies.

	Study 1		Study 2	
Measure	Pre-test *M* (*SD*)	Post-test *M* (*SD*)	Pre-test *M* (*SD*)	Post-test *M* (*SD*)
Acceptance of video games				
…in school teaching: “How much are you in favor of using video games in school teaching?”	2.90 (1.30)	2.95 (1.12)	3.63 (1.31)	3.50 (1.37)
…as a leisure activity: “How important are video games for you as a leisure activity?”	2.48 (1.50)	2.43 (1.33)	3.44 (1.82)	3.44 (1.79)
…as significant part of life: “How significant are video games in your life?”	2.10 (1.37)	2.24 (1.14)	2.94 (1.53)	3.13 (1.50)

*Values depict mean (standard deviation) of 21 biology students (Study 1) and 16 history students (Study 2).*

#### Data Analysis

For each of the following quantitative and qualitative analyses, we included all available data from each time point, while data were missing completely at random (MCAR) in both studies according to Little’s MCAR test (*p*s > 0.999).

With reference to other case studies on game-based learning in school teaching (Berg Marklund and Alklind Taylor, 2016), we conducted a thematic analysis of students’ reactions to the statements of the teacher (RQ1). We followed a theoretical/analyst-driven approach related to our research question and considered guidelines for thematic analyses (Braun and Clarke, 2006) to formulate codes and semantic themes, as illustrated by means of text excerpts.

Students’ responses during the semistructured phase of the guided student discussion (RQ1a–c) and students’ open responses in the questionnaires (RQ2a–c) were summarized and categorized following content-structured qualitative content analysis (Kuckartz, 2016). Three main categories were derived from the research questions (RQ1a = “game’s goals”, RQ1b = “game’s content”, and RQ1c = “games’ appropriateness for school teaching”), and subcategories were created based on content analysis and categorized by two independent coders. Interrater agreement was assessed in terms of Cohen’s kappa (Cohen, 1960) and was perfect in Study 1 (Cohen’s κ = 1.00) and good in Study 2 (Cohen’s κ = 0.75). Cases of disagreement were resolved through discussion.

To test potential changes in ratings over time, we used *t*-tests for dependent samples (RQ2a, RQ2b, RQ3a, and RQ4a). To test if the means of the ratings were different from the scales’ midpoints (RQ3b and RQ3c), we used one sample *t*-tests. We report effect sizes in terms of Cohen’s *d*. In line with Cohen (1988), common thresholds for small, medium, and large effect sizes are 0.2, 0.5, and 0.8, respectively. The alpha level for all statistical tests was 0.05.

### Results

#### Content of the Discussion Phase (RQ1)

The discussion phase started with the three statements of the teacher. Following the teacher’s first statement (“Playing the game Spore was fun”), the teacher asked those students with high and low ratings to elaborate on their ratings. The main theme was “game enjoyment”. One student (S5) elaborated on a high rating of 10, then a fellow student (S6) and the teacher (T) joined in:

S5: We had enough fun while, while, uh, designing our creature, simply because there were some comic factors where we then just almost fell off the chair with laughter, but…
S6: We have something funny to do right now, so…
T: I am curious.
S6: Yes, we will see (Laughter of the surrounding students).

Students talked about to continue playing, and based on the observation protocol, indeed most students would have liked to continue playing. However, some students had only limited fun gaming (ratings of 1 or 2):

S8: Because when one plays it with friends now, like in a group of four, it was a bit of fun, because we made fun of it a bit and so on. But if one imagines that one also plays it alone and in such a way—that is no fun.

Overall, students either did or did not enjoy gaming, as illustrated by the teacher’s statement: “Well, a [rating of] ten. Now we have a big emptiness, now I’m going to go there (…) [to the students with the rating] ‘I don’t agree at all’ ”. Students also discussed “game features”, in particular that the game strongly motivated to continue playing.

Following the teacher’s second statement (“The game vividly illustrates evolutionary processes”), the students were undecided. The main theme was the “implementation of evolutionary processes”:

S5: One just, I don’t know, goes there, looks for someone else from one’s species to mate with, then one modulates one’s creature as one likes. […] This means that the factor ‘mutation’ is missing.
S10: And one buys one’s characteristics. That’s not like in nature. That’s just, well, one chooses how one looks afterward, so to speak, and it’s just not like that somehow. Yes.
S11: (interjects) But how else could they have done that?
S10: (simultaneously with S12) By chance.
S5: That one randomly gets some things and later one can only choose between those things.

In the last excerpt, students first discussed evolutionary processes to be missing or unrealistic in the game but then suggested potential game changes and shifted from problems to solutions. Similarly, the theme “game character development” emerged, and students discussed both issues and solutions.

Following the teacher’s third and final statement (“The game Spore should be used in biology lessons”), students discussed the theme “game’s potential role in biology teaching”:

S18: I think it’s just fun and I think if one has fun together, then one learns better. So, if one has to make some exercise sheets all the time, I think one doesn’t learn so much, because it’s not so much fun. […]
S14: I would use it especially for younger children, because then they have a little bit of fun and learn playfully, so maybe we are already a little too old for that. […]
S5: I think that if we do it like now, for two hours in one piece, that’s okay for one lesson, I also know how it [the game] goes on. […] The first two [phases] are biological, the following ones could be directly used in politics or business [education]. These are the first two phases, which are biologically based, thereafter it’s not so good for the topic.
S22: Well, I think the game was fun, but one didn’t really learn something from it. Because what we did in class before, one learned the technical terms and how it happens, but there, that was just a game. One has not really learned anything. Yes, but as I said before, if one gets through the topic, playing something like that again at the end is like watching a film, I think it’s really good.
S8: Yes, I agree with S22 […] It would only bring me something if I should somehow find out where the evolution and the mutation and the recombination takes place and why it is like that.

To sum up, students once more discussed the relevance of game enjoyment for learning, but they also reflected on the game’s target group. A student who knew the game (S5) then argued that the gaming duration sufficed and that the game’s later phases could be appropriate for teaching other subjects. Although students thought that no topic-related learning occurred, some favored gaming at the end of a teaching unit or given topic-related search tasks.

After the statements of the teacher, the semistructured discussion phase followed. Many responses of the students to the teacher’s questions in this phase were assigned to the following subcategories: Students identified two goals in the game (RQ1a) and identified and criticized topic-related content in the game (RQ1b). When being asked to take an expert’s perspective (RQ1c), students mostly provided constructive critique and moreover discussed games’ potentials and limitations. Table 1 depicts the categories, number of statements assigned to the categories, the teacher’s questions, and example statements of the students.

#### Influence of Gaming and Discussing on Students’ Perceived Topic Knowledge (RQ2)

**FIGURE 2:**
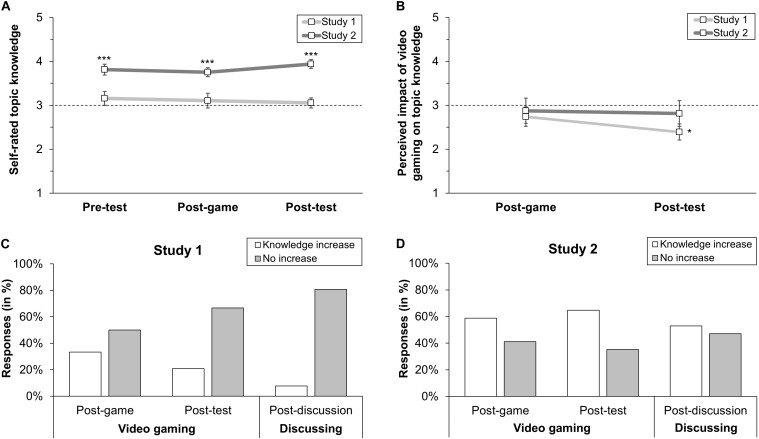
Effects of video gaming and discussing on students’ perceived topic knowledge in both studies. **(A)** Mean self-rated topic knowledge in Study 1 and Study 2. **(B)** Mean perceived impact of video gaming on topic knowledge in Study 1 and Study 2. **(C)** Open responses on perceived impact of video gaming and discussing on topic knowledge in Study 1 (missing responses not displayed). **(D)** Open responses on perceived impact of video gaming and discussing on topic knowledge in Study 2. Vertical lines indicate the standard error of the mean. Asterisks in **(A,B)** indicate the results of one sample *t*-tests comparing the means with the scales’ midpoints of 3 (dashed horizontal lines). * *p* < 0.05. ***p < 0.001.

Regarding the influence of the video gaming phase on students’ perceived topic knowledge (RQ2a), no change was found between self-rated topic knowledge at pre-test and post-game, *t*(18) = −0.44, *p* = 0.667, *d* = 0.10 (see Figure 2A). Students’ perceived impact of video gaming on topic knowledge did also not change from post-game to post-test, *t*(22) = −1.50, *p* = 0.148, *d* = 0.31 (see Figure 2B). The minority of students had the impression that playing the game increased their topic knowledge (post-game: 33.33%; post-test: 20.83%) (see Figure 2C). Further, at post-test, few students recalled one (4.17%) or three topic-related aspects (12.50%) they learned via video gaming, and about every second student did not recall anything (45.83%). The other students’ statements were about that the game was fun (8.33%), could improve skills of collaboration, communication, and tactics in general (4.17%), or disregarded topic-related content (4.17%). Of the students, 20.83% gave no open response.

Regarding the influence of the guided discussion phase on students’ perceived topic knowledge (RQ2b), we found no change in self-rated topic knowledge from post-game to post-test, *t*(19) = −0.37, *p* = 0.716, *d* = 0.08 (see Figure 2A). Students’ perceived impact of the discussion on topic knowledge was numerically slightly below the scale’s midpoint (*M* = 2.80, *SD* = 0.82), and only few students openly stated that discussing increased their topic knowledge (7.69%) (see Figure 2C).

With respect to the question whether video gaming or discussing had a stronger influence on students’ topic knowledge (RQ2c), some students indicated to have learned more through video gaming (19.23%) or discussing (11.54%), respectively. However, most responding students stated that there was no influence at all (42.31%), while some students stated a similar influence (3.85%) or gave no response (23.08%).

#### Students’ Learning Motivation Regarding Lesson’s Topic, Gaming, and Discussing (RQ3)

Measures of students’ learning motivation regarding the topic of the lesson did not change from pre- to post-test, all |*t*s| ≤ 1.92, *p*s ≥ 0.069, *d*s ≤ 0.42 (RQ3a). Table 2 shows the results in detail. Students’ game enjoyment, game interest, perceived competence, game preference for school teaching, and overall game rating as well as the perceived game’s appropriateness for school teaching were above the scales’ midpoints, all *p*s ≤ 0.005 (RQ3b). Table 3 shows the results in detail. The perceived personal relevance of the discussion was also above the scale’s midpoint (*p* = 0.025) (RQ3c). Table 4 shows the results in detail.

#### Students’ Acceptance of Video Games in School Teaching (RQ4)

Students’ acceptance of video games in school teaching did not change from pre- to post-test, *t* = 0.21, *p* = 0.833, *d* = 0.05 (RQ4a). Students’ acceptance of video games in school teaching was about the scale’s midpoint and numerically higher than students’ acceptance of video games as a leisure activity and as significant part of life (RQ4b). Detailed results are depicted in Table 5.

### Discussion

In Study 1, we integrated the commercial video game Spore into a regular double lesson of a 10th-grade Biology course, in line with the curricular topic and learning objectives. We focused on students’ reflection processes after video gaming (reflection-on-action) by means of analyzing a guided student discussion. Using text excerpts, we illustrated students’ reactions to the teacher’s three statements regarding the video game Spore. First, we found that playing Spore was fun, although we observed that students gave either very high or very low ratings. Yet, even a rather negative rating was put into perspective by saying that playing with friends, as in this case study, was fun, but playing alone would not be fun. Second, students were rather undecided regarding the statement that the game’s illustrations of evolutionary processes were vivid but gave constructive critique about how the game could be improved. The third statement suggested using Spore in biology lessons, and one student who was familiar with the game noted that prolonging the gaming phase would hardly provide additional value for biological topics. The other students’ statements indicated that students agreed with the way the game was integrated into school teaching and considered it as an object of reflection rather than a learning tool. While these three statements already triggered responses on several aspects of students’ game experiences, students raised more points during the subsequent semistructured discussion. In particular, students found that topic-related technical terms were completely missing in the game and that visual representations of evolutionary factors were simplified. In line with this, students highlighted that the game should be more realistic in terms of evolutionary theory and graphics. Students further discussed that games help in visualizing topics and can be more engaging than learning by other means of school teaching.

We also examined complementary measures of student learning, learning motivation, and acceptance of video games. Students’ self-rated topic knowledge was hardly influenced by video gaming and discussing. Open responses of the majority of students also indicated that neither video gaming nor discussing influenced the topic knowledge. Students’ learning motivation did not change regarding the lesson’s topic. Students’ game preference for school teaching and the perceived appropriateness of the game for school teaching were above the scales’ midpoints. Students’ perceived personal relevance of discussing was also above the scale’s midpoint. Finally, students’ acceptance of video games in school teaching did not change and was numerically higher than students’ acceptance of video games as leisure activity and as significant part of life.

## Study 2

Digital history games provide students opportunities to interact with numerous possible interpretations of historical narratives and events (Kee, 2011). Since a game might display only one interpretation, one should foster critical reflection in history teaching in terms of encouraging active and reflective play, treating a game critically as one interpretation, and discussing, debriefing, and evaluating games (McCall, 2016). To gain complementary results on using commercial video games in formal high school teaching, we integrated Valiant Hearts: The Great War (Ubisoft Montpellier, 2014) into a double lesson of history teaching. As in Study 1, we grounded this decision on teacher’s expertise, game reviews, a pedagogical review, and previous game-related conceptual and empirical research. Overall, both critics and user scores were positive (Metacritic, 2020b). A pedagogical review highlighted that the game deals responsibly with the topic violence and rather illustrates the fate of ordinary soldiers than showing exaggerated heroism (Spielbar, 2020b). Importantly, previous research criticized the use of stereotypes and oversimplifications in Valiant Hearts (Boltz, 2019) and outlined the importance of discussions about the game to reveal its commemorative character (Rughinis̨ and Matei, 2015). Overall, in line with curricular guidelines, we integrated Valiant Hearts into school teaching to scrutinize how students discuss the topic First World War and reflect on their game experiences.

### Materials and Methods

#### Participants

The sample comprised 17 students from a 12th-grade advanced course on history at a German comprehensive school (high school level) (*M*_age_ = 17.45, *SD*_age_ = 0.50; eight female). The 16 students present at pre-test stated to play video games frequently in their leisure time (*M* = 3.75, *SD* = 1.48, 6.25% never) but seldom in school teaching (*M* = 1.63, *SD* = 0.62; 43.75% never), about the First World War (*M* = 2.13, *SD* = 1.31, 50% never), and the game Valiant Hearts (*M* = 1.56, *SD* = 1.26, 81.25% never, 6.25% occasionally, 6.25% frequently, and 6.25% very frequently).

#### Design and Procedure

The design and procedure were identical to that of Study 1, except that the teacher assigned students to groups of two. The teacher ensured that each student played for a similar duration (20 min). We collaborated with a history teacher with personal interest in video games and some experience with using video games in school teaching. The collaboration with the teacher was initiated by the same announcement as in Study 1. The first author used the same observation protocol as in Study 1 and noticed technical issues at the beginning of the first lesson causing a short delay. During video gaming, students’ conversations were about the puzzles in the game, and the teacher or students occasionally provided a hint to groups unable to solve a puzzle for a while.

#### Teaching Materials

We developed similar teaching materials as in Study 1, in close collaboration with the teacher. We used Valiant Hearts: The Great War (Ubisoft Montpellier, 2014), a single player adventure and puzzle video game dealing with historical events of the First World War (age rating of 12). The game sequence comprised the declaration of war, the beginning of the war (from the perspective of a French soldier), the First Battle of the Marne (from the perspective of an US-American soldier), and the Battle of Neuve Chapelle (again from the perspective of the French soldier). To proceed in the game, players have to solve puzzles and interact with non-player characters. At various points in the game, players can access optional textual and pictorial information about facts and items related to the First World War. Each group of students played the game using a mobile tablet and in-ear headphones.

#### Measures

The measures were almost identical to Study 1, except from the teacher’s statements and topic-related items. The teacher stated the following three statements: “The game is boring”, “Comic drawings do not do justice to the seriousness of the topic”, and “The game Valiant Hearts does not belong to history lessons”. During the subsequent semistructured discussion, the teacher asked one question about the game’s goal (RQ1a), three questions addressing the game’s content (RQ1b), and one question addressing games’ perceived appropriateness for school teaching (RQ1c). Question wordings are depicted in Table 6. Unlike in Study 1, we analyzed four additional questions that the history teacher asked: “Which emotions does the game arouse, and by what?”, “Does the game use stereotypes, and what for?”, “How does the game position itself to violence and the experience of violence?”, and “What historical narrative does this game convey?”. We reformulated topic-related items by replacing the term “evolution” by “First World War”.

**TABLE 6:** Content of the semistructured discussion phase in Study 2.

Category and subcategories	*n*	Teacher’s questions and students’ example statements
**Game’s goals**		What is the general goal of the game? What has allowed or hindered you to reach it?
Knowledge transfer	2	“Well, I think there were multiple goals. On the one hand, that the historical context was represented and taught” (S5) “The goal of the game was simply to play through the time from 1914 to 1918 and to collect general information about everyday life, about that time, that year, so that one deepens one’s knowledge and also learns something new” (S2)
Survival	1	“I think the goal of the game was also simply to survive, because one faces many situations throughout the game, where one, I’d say, would simply die” (S4)
Change in perspective	2	“That one gets a different point of view in the game” (S9)

**Game’s content**		At which points did the game, in favor of gameplay, represent historical events in an altered or simplified manner? Were there game scenes, in which historical events were perhaps even represented incorrectly? What perspective does the game take?
Content-based criticism	1	“So, the declaration of war was actually not explained in detail” (S10)
Content simplification via gameplay	3	“It was displayed in the upper right corner when the enemy was reloading the weapon, so that one knew when and when not to run” (S6)
Content representation	3	“I think it is good that the perspective from a normal person was taken. That could have been absolutely anybody during the First World War, with respect to the three perspectives taken in the game, and not a glorious hero who rushes over the battleground annihilating everything” (S4)

**Games’ appropriateness for school teaching**		Which changes would you make to the game if you were an historian?
Constructive critique	9	“I think that these historical aspects, one can basically read in the book, one can simply click away. […] It could be explained using cutscenes, so that in case of events one does not have to read” (S11) “Putting everything into a game—that would go beyond its scope. Because one cannot create a scene that takes an hour to understand the whole context” (S12) “I would change the perspective somewhat, such that we look at it differently and that we cannot just run forward or run backward. Maybe that one can see the crowds of people from above […] So, I forget that the masses of people who died, ran toward each other, and killed each other, that has not become so clear” (S10) “If you find a mixture, so that it is graphically good, that one also wants to play this game, and this information is still in there, then one could learn the topic quite well” (S2)
Games’ limitations	2	“I think one cannot learn enough with such a game, because in our first exam, we wrote a lot about the reasons, which were contained too little to feel well prepared and to take the exam after playing the game, what is not really the point of playing it, but yes” (S12) “I think it is perhaps not so suitable that one would be prepared for an exam. […] Maybe to get started, but not *per se*” (S14)

*Categories and example statements are based on responses of 9 students, while in total, 17 students participated in the discussion.*

### Results

#### Content of the Discussion Phase (RQ1)

The discussion phase started with the three statements of the teacher. Following the teacher’s first statement (“The game is boring”), only few students agreed. The first themes that emerged were “game’s narrative” and “game enjoyment”:

S1: Yes, I don’t know, this is only from a French point of view I would say. […] And that’s always just from left to right, so, I didn’t find it so exciting.
S12: Yes, actually I agree with S1.
T: Then, why don’t you stand at seven or eight?
S12: Because in a certain way it was not that boring, because one had to do something. It wasn’t that I just watched a film that might be boring […]

In regard to this first excerpt, S1 criticized the game’s narrative in terms of a rather one-sided perspective and reported little game enjoyment. After the teacher (T) questioned why S12 agreed with S1 while providing a lower rating (7.5 versus 5), S12 elaborated on game enjoyment and used a media comparison (games versus films) to outline that games allow for active usage. Students with ratings close to zero continued discussing:

S2: I found the game relatively good for learning, still now. Because time also simply passed now much faster than usual, simply because it was more interesting to play through it just like that. […] But now we already knew a lot about the topic anyway, so I don’t think it expanded our knowledge. But if we had done that at the beginning, so, also played at home in leisure time, then one could have taken something from that.
S3: Well, I thought that it was actually fun, that controls were limited, and one actually had a mission what to do. And it was actually fun (Teacher: Nice). So, for the fact that I am not a computer fan.
S4: I really liked the game. I also thought it was really good for learning. But I also looked at the entries, I skimmed over them, so I didn’t just leave them out completely. […] Because these entries were shown exactly when one asked oneself questions. […]
S5: I wanted to add that the game can, I would say, also be played to the end, that is, to the conclusion. I think it’s also good to, I’d say, refresh one’s knowledge a bit, so that it gets reactivated. But I also think that some details have been mentioned that were perhaps not discussed in class.

Regarding the last excerpt, the theme “game for learning” occurred several times: S2 suggested that starting a teaching unit with this game could increase topic knowledge, S4 outlined the educational value of the contextual yet optional information provided by the game, and S5 argued how the game could be beneficial for learning in several ways. Related to the theme “game enjoyment”, S2 referred to other school lessons and felt as if time passed faster because gaming was more interesting. As a non-gamer, S3 agreed and favored the simple controls and that goals of the game were clear.

Following the second statement of the teacher (“Comic drawings do not do justice to the seriousness of the topic”), one student (S5) was undecided (rating of 5):

S5: Because I think that although the music that was played there did indicate a certain seriousness, I don’t think that the drawing itself can really convey this, that is to say, convey it to this extent, as it actually was or as we had now discussed.
T: Comic drawings do not do justice to the seriousness of the subject. The others all stand there almost at zero.
S6: One also has to remember that the game is played on a tablet and possibilities are not so huge for high-end graphics. […]
S4: I think the graphic style didn’t really influence that at all, because I think the colors, they were pretty gray and pretty sad. It was always like that, very serious and the comic graphics didn’t really distract me. […] The comic graphics don’t play such a role when the colors are quite dark, and the music is really sad.

In sum, the students did not consider the comic style as inappropriate to display the topic, as emphasized by the teacher’s statement. Students discussed the main theme “game graphics” and considered that graphics might also be simplified so that the game can be used on devices with less processing power (such as tablets). Students moreover outlined that the seriousness of the topic was mainly conveyed via the music and the color scheme of the game.

Following the teacher’s third and final statement (“The game Valiant Hearts does not belong to history lessons”), the students discussed several aspects:

S7: Yes, in my opinion it just shows the different points of view of the different soldiers. It wasn’t just France now, there was also Germany at the end, so if one had continued playing that, the USA was there of course. […] So, I think that fits in there quite well.
S3: I also found the side information to be very informative, because one doesn’t discuss everything in history class.
S8: I think one can really learn something from that. Well, first of all, we have the topic and so I think, one can play such a game now and then.

To sum up, students again discussed the themes “game’s narrative” in terms of multiple viewpoints (S7) and “game for learning” in terms of the game’s learning content (S3) and curriculum relatedness (S8). Overall, students found the game appropriate for history lessons.

Following the teacher’s statements, the teacher continued to ask questions regarding the game’s goals (RQ1a), game’s content (RQ1b), and games’ appropriateness for school teaching (RQ1c). As in Study 1, we summarized students’ answers by means of subcategories and example statements, see Table 6: Students discussed knowledge transfer, survival, and change in perspective as goals of the game (RQ1a) and discussed the game’s content in terms of content-based criticism, content simplification via gameplay, and content representation (RQ1b). Concerning games’ appropriateness for school teaching (RQ1c), students mainly provided constructive critique and discussed limitations, in particular that playing the game would not suffice to prepare for the regular course exam.

Then, the teacher continued to discuss the additional questions on emotions, stereotypes, violence, and the game’s narrative, for each of which we identified themes discussed by the students. Regarding the teacher’s question on emotions (“Which emotions does the game arouse, and by what?”), students discussed the theme “inappropriate emotions”:

S3: It wasn’t really conveyed that it [the topic] is that serious and that it was supposed to evoke grief […]
T: Okay. So, the emotions that you actually expected, they weren’t there?
S3: Yes.
S12: I thought the game actually evoked the wrong emotions. I think one gets a lot of fun and one thinks ‘yeah, cool’, but that’s just not what the First World War was. It’s about that one doesn’t really understand, sometimes not at all, which emotions are supposed to emerge, but simply this grief or despair or so. […]
S9: So, I agree with S12, because when I had the conversation [in the game], there you [the observing student] also felt like beating up the one there or, I don’t know, throwing a stick of dynamite somewhere or something. And, well, this is conveyed in a completely different way, as if one was in the mood for war, but this [the game] is actually only meant to show how bad it was back then. But that doesn’t come across when one plays it. […]
S5: Even if the emotions were not the right ones, the music and certain scenes that were being played back, where one couldn’t play—the cutscenes—emotions are rather shown in that way.

In relation to the last excerpt, S3 experienced different emotions during gaming than expected regarding the topic. S12 and S9 once more articulated the theme “game enjoyment” and that gaming evoked fun instead. With regard to the topic, this could limit the understanding (S12) or convey an inappropriate mood (S9). Finally, S5 agreed but highlighted that the music and cutscenes of the game also communicated emotions. The theme “reflections on game experiences” also emerged:

S4: I think one has to differentiate very strongly between what one feels when one plays and what one feels when one really thinks about what actually happened. Well, games should be fun, that’s the point of it, it’s a medium of entertainment. If a game would not be fun, nobody would play it, that is logical. […] And if one just thinks about what just happened, what one did, for just a second, then it can bring about grief and a bit of pity, if one really thinks about what just happened. And one didn’t really notice that people died there, for example, when one ran along there, because one was just concentrated on surviving and that was mostly the case during the war. But when one has thought about it afterward, one feels a little sad. […]
S12: Well, say I’m talking to S2 about it [the game] and then, uh, we wouldn’t both start reflecting on it, but just say, ‘yes, I killed that one, haha, it was really funny, I kind of knocked him out with one punch’. I don’t think that we teenagers or in general one would sit down there and think about it all over again. […] I don’t think that one would really become aware of it, because that’s just, they all say ‘it’s just a game’, so, I don’t have to die for real.

Summing up, S4 made the point that it is important to distinguish between emotions during gaming and after gaming. S12 then outlined a hypothetical example, which indicates that teenaged students might not exchange serious considerations after gaming (reflection-on-action) on their own.

Regarding the teacher’s question on stereotypes (“Does the game use stereotypes, and what for?”), students first named stereotypic properties of game characters (e.g., “wine” and “baguette” were related to French people). Then, the teacher elaborated on the original question: “One would not have had to do it. What does the game do that for? Why do the developers do this?”, and students discussed:

S2: In any case, it also clarifies the character of these, of these squads, by somehow bringing in this French war music, or so, so one notices that these are the French.
S6: I think the developers have done that to make it a bit more humorous. I don’t think it was about making the Germans look more like Germans. I think the game did that quite well.
S4: One also has to consider that the game almost contains no spoken language. […] And since the game should also work for younger people, […] one has to make it clear that even someone who doesn’t know about history knows ‘I see, they are Germans, oh, now I’m going to play a French’ — that this is also correctly understood. And also to make it more humorous, I also agree with that.

In sum, the students discussed the theme “functions of stereotypes”. They mentioned that stereotypic properties facilitated to correctly identify game characters’ nationalities (e.g., French, German, and US-American), which could support younger people and people without history knowledge to understand the game’s narrative. Students also discussed that stereotypes can add some humor.

Following the teacher’s question on violence (“How does the game position itself to violence and the experience of violence?”), the students discussed:

S6: One only has to knock them out, the goal is just to get through the game with as little violence as possible. That makes the game pretty good. […]
S2: If one wants to teach the eighth graders something and then somehow, I don’t know, bring something really brutal into it and make the graphics realistic, then I think they are really disturbed and don’t come to school […] or some of them prefer to come to school, that’s also possible. […]
S4: One should keep in mind that although one doesn’t use violence oneself, not really, one’s opponents indeed do. That they, they really come with machine guns and bombs.

To summarize, the main theme was “elements of violence”, and students mentioned that there was relatively little violence in the game and that, as players of the game, one used little violence. Students also discussed the theme “effects of violence” and that adding more brutality and more realistic graphics to the game could negatively affect younger students.

Regarding the teacher’s question on the game’s narrative (“What historical narrative does this game convey?”), the teacher also asked students to relate the game’s content to content of previous lessons:

T: Can you relate this to the graves and monuments, with which monument would you more likely associate this and with which not at all? Why?
S4: Well, I think, when one plays the game, one should also make one’s own opinion of how one thinks it is. And about the monuments, I think of the monuments that we looked at in class, it doesn’t fit so well, because the monuments always made it very clear that war is something good or bad. […]
S1: I think that the [monument of a] mourning woman fits quite well. Because in the beginning one sees how the family is torn apart and how they have to stay alone. So, I think it fits, because the monument was aimed at all nations. And I think that in turn also fits into the game.

To sum up, the theme “normative ethics” emerged, while students did not think that the game clearly communicated whether war was good or bad. They also discussed the theme “related course content” by referencing the game’s narrative to monuments the students had discussed in previous lessons.

#### Influence of Gaming and Discussing on Students’ Perceived Topic Knowledge (RQ2)

Regarding the influence of the video gaming phase on students’ perceived topic knowledge (RQ2a), there was no change in students’ self-rated topic knowledge from pre-test to post-game, *t*(15) = −0.44, *p* = 0.669, *d* = 0.11 (see Figure 2A). Students’ perceived impact of video gaming on topic knowledge was numerically slightly below the scale’s midpoint at post-game and post-test (see Figure 2B). Students’ open responses revealed that playing the game increased most students’ perceived topic knowledge (post-game: 58.82%; post-test: 64.71%) (see Figure 2D). Based on the free recall, students remembered one (47.06%), two (17.65%), or three topic-related aspects (17.65%) they learned by playing the game; the other students stated that playing motivated for further investigations (5.88%) or could avoid boredom (5.88%). Of the students, 5.88% gave no response.

Regarding the influence of the discussion phase on students’ perceived topic knowledge (RQ2b), we found no change in self-rated topic knowledge from post-game to post-test, *t*(15) = 1.86, *p* = 0.083, *d* = 0.47 (see Figure 2A). Students’ perceived impact of the discussion on topic knowledge was numerically slightly below the scale’s midpoint (*M* = 2.71, *SD* = 0.92). About half of the students (52.93%) stated that discussing increased their knowledge in terms of an exchange of player perspectives (29.41%), content-related depth (11.76%), and reactivation of knowledge (11.76%); the other students (47.06%) stated no knowledge increase (see Figure 2D).

At post-discussion, a higher number of students indicated to have learned more through video gaming (58.82%) than through discussing (11.76%), while the other students stated a similar influence (11.76%) or no influence (17.65%) (RQ2c).

#### Students’ Learning Motivation Regarding Lesson’s Topic, Gaming, and Discussing (RQ3)

Measures of students’ learning motivation regarding the lesson’s topic did not change from pre- to post-test, all |*t*s| ≤ 1.46, *p*s ≥ 0.164, *d*s ≤ 0.37 (RQ3a) (see Table 2). Students’ game enjoyment, perceived competence, and all satisfaction measures were above the scales’ midpoints (all *p*s ≤ 0.037) (RQ3b) (see Table 3). Students’ personal interest and personal relevance of discussing was also above the scales’ midpoints (all *p*s ≤ 0.001) (RQ3c) (see Table 4).

#### Students’ Acceptance of Video Games in School Teaching (RQ4)

Students’ acceptance of video games in school teaching did not change from pre- to post-test, *t* = −0.81, *p* = 0.432, *d* = 0.20 (RQ4a). Students’ acceptance of video games in school teaching was numerically higher than the scale’s midpoint and numerically higher than students’ acceptance of video games as a leisure activity and as significant part of life (RQ4b). The detailed results are depicted in Table 5.

### Discussion

In Study 2, we integrated the commercial video game Valiant Hearts: The Great War (Ubisoft Montpellier, 2014) into a regular double lesson of a 12th-grade advanced course on history, in line with the curricular topic and learning objectives. The reflection processes of the students were triggered by the teacher’s three statements about the game experiences. First, a student found the game rather boring due to a one-sided narrative, while other students discussed several reasons why they were excited by playing the game: time went by faster, controls were simple, the game’s goals were clear, and the game provided contextual learning content. Second, being asked about an illustrative feature of the game (comic drawings), most students found this feature appropriate to convey the serious topic of the First World War and outlined that the music and the color scheme of video games also contribute to an overall game experience. Third, students rather argued in favor of Valiant Hearts belonging to history lessons, since the game shows different viewpoints, provides topic-related information, and the students saw an overall educational value of playing such games occasionally in school teaching. During the subsequent semistructured discussion, students criticized the events displayed in the game as partially imprecise or simplified, found that historical facts were missing, that attending the learning content is optional in the game, and that playing such games could not suffice for exam preparation. In turn, they discussed that particularly important facts should become mandatory to prevent that players are missing those.

In reaction to the teacher’s additional question on emotion, students discussed how different multimedia features of the game evoked emotions and raised two important points: On the one hand, students reflected on the game being an entertainment product that evoked fun as a positive emotion, instead of the expected negative emotions related to the First World War—such as grief and despair. On the other hand, another student outlined the importance to distinguish between emotions during gaming and after gaming, similar to the key differentiation between reflection-in-action and reflection-on-action that we introduced in this case study. Moreover, a student argued that reflection might not even take place by itself and without reflection triggers, which is in line with findings on how players reflected on game experiences from leisure time (Mekler et al., 2018). Stereotypes are part of the game (Boltz, 2019), and students discussed two functions: Stereotypes could facilitate correct assignment of game characters to groups or nationalities and might add some humor to the game. Students also realized and favored that the game does not endorse violence, since players make significantly less use of violence than players’ opponents. Finally, evaluating the game’s narrative allowed students to reactivate knowledge by relating the game’s content to previous lessons’ content. Overall, while students actively discussed multiple relevant aspects related to the game after playing it, we would like to highlight that students themselves pointed out the value of such a discussion phase, also since reflection might not take place by itself and not during gaming.

Regarding student learning, students’ perceived topic knowledge was hardly influenced by video gaming and discussing. Still, most students openly stated that the game increased their topic knowledge, and most students recalled one to three topic-related aspects they learned through gaming. Students furthermore stated that discussing allowed for exchanging player perspectives, content-related depth, and reactivating knowledge. More students stated that they learned more through video gaming than through discussing. Students’ learning motivation regarding the lesson’s topic did not change. Students’ ratings for playing the game again in school teaching and game’s appropriateness for school teaching were above the scales’ midpoints, while qualitative results suggest that some students doubted the game’s appropriateness for school teaching. Students’ personal interest as well as relevance of discussing were also above the scales’ midpoints. Finally, students’ acceptance of video games in school teaching did not change and was numerically higher than students’ acceptance of video games as leisure activity and as significant part of life.

## General Discussion

It has been discussed for years *that* commercial video games can be used for learning (Boyle et al., 2016; Clark et al., 2016) and for formal education (Gee, 2005; Squire, 2008; van Eck, 2009; Hainey et al., 2016), while our case studies addressed the current need for more research on *how* to integrate these games into formal educational settings (Mayer, 2019). Our case studies illustrate how commercial video games might serve as objects of reflection in formal school teaching, in line with the curricular topic and learning objectives: Student learning and reflection can be guided by means of worksheets during a gaming phase, and a following discussion phase moderated by a teacher can allow students to critically discuss and reflect on their game experiences. With regard to this focal question, we next condense the students’ reflections on their game experiences to formulate several suggestions in regard to using commercial video games as objects of reflection.

Concerning student reflection, we found that some central statements of a teacher about the quality of the game can already trigger student reflections, for instance reactions on the games’ level of entertainment, their appropriateness of visualizing a lesson’s topic, and their suitability for teaching. More specifically, students may discuss multiple goals of the games (RQ1a), how the games represent learning content (RQ1b), and evaluate the game from an expert’s perspective (RQ1c). First, students can identify multiple goals in games so that one could trigger and guide their reflections not only on different strategies of playing games (Kiili, 2007) but also on relations between their game experiences and real events, behaviors, or decisions. Reflective guidance (e.g., by means of verbal instructions and worksheets as in our studies) can support students to pursue learning objectives instead of performance goals or no goals, which may reduce task complexity and increase fun in game-based learning contexts (Nebel et al., 2017). Second, playing and discussing games in school teaching can allow students to share experiences on whether and how games represent content related to a subject matter in a simplified way. Third, rather than relying on individual game experiences, playing games in school teaching can allow students to formulate, discuss, and reflect on constructive critiques and own ideas for modifications of the games together with fellow students and teachers. That most of the students’ statements belonged to this category also points to the potential of letting students design own games (Prensky, 2008; An, 2016) as well as to the benefits of perspective taking and using games as “objects-to-think-with” (Dishon and Kafai, 2020, p. 1). Notably, it seems valuable to also address more general topics such as emotions, stereotypes, violence, and narratives when discussing video games. More generally, we may speculate that student discussions could foster students’ media literacy in terms of procedural knowledge, that is, how to analyze commercial video games’ goals and content and how to identify their potentials and limitations for learning. Taken together, we conclude that integrations of commercial video games into formal educational settings could include a subsequent discussion phase to let students share game experiences and reflect on the games’ goals, content, and appropriateness for school teaching.

Concerning student learning, only qualitative results of Study 2 provided partial evidence that video gaming (RQ2a) and discussing (RQ2b) increased students’ topic knowledge. Furthermore, only in Study 2 students stated that they learned more through video gaming than through the discussion (RQ2c). Overall, while our non-significant quantitative results are in line with previous meta-analytic findings suggesting single play sessions being insufficient to increase learning outcomes (Wouters et al., 2013; Clark et al., 2016), mainly the qualitative results of Study 2 offered partial evidence that a single play session, followed by a guided student discussion, could increase students’ topic knowledge. Moreover, most corresponding effect sizes were small in both studies. Only in case of the non-significant influence of the discussion phase on students’ self-rated topic knowledge in Study 2, the effect size was moderate. This might partly rely on the game itself or on the fact that the discussion contained additional questions on several game- and content-related topics, as compared to Study 1. While in Study 1 more students responded that gaming influenced their topic knowledge after gaming than four days later (post-game versus post-test), in Study 2, the responses were more stable across time. Notably, students in both studies found the games appropriate for school teaching, whereas students in Study 2 noted that playing the game might not suffice for exam preparation. Taken together, guided discovery learning through video gaming alone seems to hardly benefit students’ topic knowledge in formal educational settings.

Concerning students’ learning motivation, we considered main factors of learning motivation (Keller, 2016) regarding the lessons’ topics, video gaming, and discussing. First, students’ learning motivation regarding the lessons’ topics did not change (RQ3a), which relates to other studies in which single play sessions did not change students’ motivation (Wouters et al., 2013). Second, students’ learning motivation regarding video gaming was relatively high in both studies (RQ3b) in terms of game enjoyment, perceived competence, game interest, game preference for school teaching, and games’ appropriateness for school teaching. In specific, high game enjoyment indicates that students were intrinsically motivated, and high perceived competence indicates that students were able to handle the games’ challenges and controls (Ryan et al., 2006). Third, students’ learning motivation regarding discussing (RQ3c) was about the scales’ midpoints in Study 1 and above the scales’ midpoints in Study 2. Overall, our results do not show that our way of using and discussing commercial video games for a double lesson changes students’ learning motivation but could allow students to experience high enjoyment, competence, and satisfaction.

Concerning students’ acceptance of video games, we provide results from the context of use that are often lacking in studies on video game acceptance (Bourgonjon et al., 2010; Wang and Goh, 2017). In our studies, students were undecided (Study 1) or tended to accept video games in school teaching (Study 2), while acceptance in school teaching was highest (versus as leisure activity and as significant part of life) (RQ4). Considering that students gave high ratings for game preference for school teaching and game’s appropriateness for school teaching (RQ3b), it overall seems that students could welcome the use of commercial video games in school teaching.

Taken together, our case studies demonstrate *how* commercial video games can be used as objects of reflection in formal educational settings. Importantly, we employed mixed methods to thoroughly investigate how playing and discussing commercial video games in formal educational settings might affect learning and reflection processes. Our results indicate that at least some commercial video games are ineffective in disseminating declarative knowledge and should not be regarded nor be used as content providers. Rather such commercial video games could serve as objects of reflection in formal educational settings, for instance, by having teachers moderating student discussions on games’ goals, content, and appropriateness for school teaching. Considering that teachers with gaming experience and technological competence can facilitate successful integrations of commercial video games (van Eck, 2009), other commercial video games could be used in similar ways to teach evolution (Leith et al., 2016), history (Schrier, 2014), or other topics of formal education. While commercial video games are not frequently used in high school teaching to teach twenty-first century skills (Qian and Clark, 2016), more such integrations could allow to foster students’ reflection processes or related critical thinking skills. Overall, appropriate uses and discussions of video games could facilitate to meet the media reality of many students as well as to foster key media literacy skills (Squire, 2008).

### Limitations and Future Research

Both presented case studies are conceptually similar and provide results from a basic (Study 1) and advanced high school course (Study 2). Both case studies were of a specific duration (one double lesson) and contained participants in a specific age range (15- to 18-year-olds). We moreover investigated convenience samples with rather small sample sizes. However, we would like to highlight that our samples reflect sizes of regular school classes and that effects should also be detectable at the level of school classes to be of practical relevance. Although our measures were not standardized, we used a rigorous translation–back translation process to transform validated scales into direct rating scales, resulting in acceptable internal consistency in terms of Cronbach’s alpha (Tavakol and Dennick, 2011). Still, our results do only rely on students’ self-reports and do not include objective test results such as, for instance, results from a test on topic knowledge or on media literacy competence. While we triangulated qualitative and quantitative methods and data, we did not consider any teacher ratings nor in-game measures of student learning. Overall, limited generalizability and comparability of related results in this research field is a common yet critical problem and partly can be traced back to the issue of a missing unequivocal terminology (cf. Rüth and Kaspar, 2017). In this regard, there may be conceptual overlaps between the terms commercial video games and serious games, the latter aiming at maximizing learning and motivation effects by blending entertainment and learning (Breuer and Bente, 2010). Indeed, while the appropriateness of the term serious game is a matter of debate in game science (Klabbers, 2018), in practice, one can identify aspects that commercial video games share with serious games (Ulrich and Helms, 2017) and assess their educational value (Becker, 2017a; Rüth, 2017). Using this rationale, we noted in advance that learning from the game in Study 1 is problematic due to its biased representations of evolution (Schrader et al., 2016), but guided learning with the game is conceivable (Schrader and McCreery, 2012). Similarly, we noted that the game we used in Study 2 was a commercial video game that contained optional textual and pictorial information related to the topic. In this sense, Study 2 was not only complementary in terms of investigating another topic and a more advanced course but also in terms of using a commercial video game with more elaborate learning features than the game used in Study 1. Similarly, future research might benefit from evaluating games’ features to further examine commercial video games’ roles and effects.

To scrutinize how commercial video games could be best integrated into formal school teaching and other formal learning settings, further research is needed. Future investigations could go beyond the dimension of reflection-on-action and use other measures of reflective learning (Brockbank and McGill, 2007a) to provide more insights, for instance, on different levels of reflection processes in game-based learning (Mekler et al., 2018). Such investigations could also evaluate effects based on intermediate results such as game scores (formative evaluation) or based on final results such as the self-reports in our pre–post design (summative evaluation). Overall, several measures before, during, and after gaming as well as other aspects of the study design can be considered (e.g., All et al., 2016; Rüth, 2017). Measurement time points yet should be chosen carefully, and future studies could use measures at different time points, for instance, to investigate whether and how the perceived influence of gaming on learning changes over time. To test appropriate instructional approaches, one could adapt guidelines for lesson plans, for example, on teaching evolution (Nelson, 2012; Southerland and Nadelson, 2012) or history (McCall, 2016; Carvalho, 2017). Notably, several studies on game-based learning are missing links to a learning theory (Wu et al., 2012) so that future research could aim at supplementing video gaming with another instructional method (such as the guided discovery learning of our case studies) and let students work in groups, both being ways to increase learning (Wouters et al., 2013). However, regarding instructional guidelines, a specific learning instruction might not be beneficial for learning when using commercial video games (Hawlitschek and Joeckel, 2017). Future studies could adapt instructional approaches across multiple lessons and investigate longitudinal effects of video gaming and discussing on student reflection as well as student learning and motivation. While we focused on students’ statements and self-ratings, one could also investigate how students create and share artifacts (e.g., screenshots or notes) and trace players’ hypotheses and decisions more precisely (Schrader and McCreery, 2012). Further, such subjective measures could be complemented with teacher ratings of student performance and objective test results as well as in-game metrics. For instance, students’ self-reported dance skill and game score were found to change differently over time across four game-based lessons in a formal educational setting (Rüth and Kaspar, 2020). One could also ask teachers to think aloud, for instance, retrospectively on their experiences about teaching with the game or concurrently when playing a game to explore how they evaluate its educational value. Furthermore, video gaming can be integrated into formal education via digital environments as in flipped classroom settings (e.g., students play games at home and discuss them in the classroom) or distance learning settings (e.g., teachers guide game sessions or discussions via video conferences). One could then examine video recordings from such settings, for instance, to investigate the quantity and quality of student–student or teacher–student interactions and to compare those between game-based and non-game settings. Finally, there are other lines of game-based research (cf. Mayer, 2019), and one might compare how students reflect on video games containing different features or on video games and other media, such as interactive e-books or interactive videos.

To conclude, we presented two case studies to demonstrate how commercial video games could be integrated as objects of reflection into formal school teaching. We illustrated how students reflected on their game experiences and on commercial video games’ goals, content, and appropriateness for school teaching. It overall seems that teachers who provide students guidance during gaming and discussing allow students reflection processes that might foster their media literacy skills. In this regard, using commercial video games as objects of reflection appears to be a promising approach to foster student reflection in future formal education.

## Data Availability Statement

The raw data supporting the conclusions of this article will be made available by the authors, without undue reservation.

## Ethics Statement

Ethics board approval was not required for the study on human participants in accordance with the local legislation and institutional requirements and as the video games’ integration into school teaching was in accordance with the formal school curriculum. Written informed consent to participate in this study was provided by the students’ legal guardians/next of kin and all students voluntarily participated in this study.

## Author Contributions

MR and KK designed the study and interpreted the results. MR conducted the literature review, organized and supervised the data collection, performed the statistical analyses, and drafted and revised the manuscript. KK supervised the statistical analyses, revised the manuscript, and acquired the funding. Both authors contributed to the article and approved the submitted version.

## Conflict of Interest

The authors declare that the research was conducted in the absence of any commercial or financial relationships that could be construed as a potential conflict of interest.
