# Supplementary material for: Commercial Video Games in School Teaching: Two Mixed Methods Case Studies on Students’ Reflection Processes
Source: Front Psychol. 2021 Jan 26;11:594013. doi: 10.3389/fpsyg.2020.594013 (PMC7870496; doi:10.3389/fpsyg.2020.594013)
Supplement: Supplementary file 1 [file Table_1.pdf]

Supplementary Materials

Supplementary Table 1. The complete measurement plan of Study 1 and Study 2.

RQ	Measure	Pre-test	Post-game	In-discussion	Post-discussion	Post-test
RQ1	Guided student discussion			O		
RQ1a	… on game's goals[a]			O		
RQ1b	… on game's content[a]			O		
RQ1c	… on games' appropriateness for school teaching[a]			O		
RQ2a	Influence of gaming on students' perceived topic knowledge					
	Self-rated topic knowledge[b]	O	O			
	Perceived impact of video gaming on topic knowledge self-rated[c], open response[d]		O			O
	Free recall[d]					O
RQ2b	Influence of discussing on students' perceived topic knowledge					
	Self-rated topic knowledge[e]		O			O
	Perceived impact of discussing on topic knowledge self-rated[f], open response[a]				O	
RQ2c	Perceived impact of video gaming compared to discussing on students' perceived topic knowledge[a]				O	
RQ3a	Students' learning motivation regarding lesson's topic					
	Topic interest[g]	O				O
	Topic commitment[g]	O				O
	Personal relevance of the topic[g]	O				O
	Social relevance of the topic[g]	O				O
	Volition to learn about the topic (three items)[g]	O				O
RQ3b	Students' learning motivation regarding video gaming					
	Game enjoyment (four items)[a]		O			
	Game interest[a]		O			
	Perceived competence (five items)[a]		O			
	Satisfaction: Game preference					
	Game recommendation[a]		O			
	Game preference for school teaching[a]		O			
	Game preference in leisure time[a]		O			
	Satisfaction: Game evaluation					
	Game graphics[a]		O			
	Game music[h]		O			
	Overall game rating[i]		O			
	Game's appropriateness for school teaching[a]		O			
RQ3c	Students' learning motivation regarding discussing					
	Personal interest in the discussion[a]				O	
	Personal relevance of the discussion[a]				O	
	Motivation to participate in the discussion[e]				O	
RQ4	Acceptance of video games in school teaching[g]	O				O
	Acceptance of video games as a leisure activity[g]	O				O
	Acceptance of video games as significant part of life[g]	O				O

Note. Different sample sizes are due to students being absent at the respective times or data missing completely at random (MCAR) in both studies, according to Little's MCAR test ($ps > 0.999$). Pre-test: the day before the lesson; post-game: following video gaming; in-/post-discussion: during/following the discussion; post-test: at the beginning of the following lesson that was four days later.

[a]$n = 26$ (Study 1), $n = 17$ (Study 2); [b]$n = 19$, $n = 16$; [c]$n = 23$, $n = 16$; [d]$n = 24$, $n = 17$; [e]$n = 20$, $n = 16$; [f]$n = 25$, $n = 17$; [g]$n = 21$, $n = 16$; [h]$n = 15$, $n = 16$; [i]$n = 26$, $n = 16$.
